# Preparation of hydroxy genkwanin nanosuspensions and their enhanced antitumor efficacy against breast cancer

**DOI:** 10.1080/10717544.2020.1770372

**Published:** 2020-06-03

**Authors:** Hui Ao, Yijing Li, Haowen Li, Yian Wang, Meihua Han, Yifei Guo, Rongxing Shi, Feng Yue, Xiangtao Wang

**Affiliations:** a Institute of Medicinal Plant Development, Chinese Academy of Medical Sciences & Peking Union Medical College, Beijing, PR China; b China-Japan Friendship Hospital, Bejing, China; c Guangdong Jiabo Pharmaceutical Co., Ltd., Guangdong, People’s Republic of China

**Keywords:** Hydroxy genkwanin, nanosuspensions, cytotoxicity, antitumor efficacy, breast cancer

## Abstract

Hydroxy genkwanin (HGK), a flavonoid compound from natural resources, showed good inhibition against the growth of breast tumor cells. However, the poor solubility restricted the further study and the *in vivo* drug delivery of HGK. We prepared HGK nanosuspensions by antisolvent precipitation method and investigated their characterization, stability, hemolysis probability, release behavior *in vitro*, antitumor activity *in vitro* and *in vivo*, and preliminary safety through acute toxicity experiments. The resultant HGK nanosuspensions (HGK-NSps) showed an average diameter of (261.1 ± 4.8 nm), a narrow particle size distribution (PDI of 0.12 ± 0.01), spherical morphology, high drug-loading content (39.9 ± 2.3%, w/w), and good stability in various physiological media. HGK-NSps was safe for intravenous injection at low concentration and HGK was slowly released from the obtained nanosuspensions. HGK-NSps showed stronger cytotoxicity than free HGK against many tumor cells *in vitro*. Especially against MCF-7 cells, the IC_50_ value was decreased to 1.0 μg/mL, 5-fold lower than the HGK solution. In the *in vivo* antitumor activity study HGK-NSps (40 mg/kg) displayed a similar therapeutic effect to that of the paclitaxel injection (8 mg/kg). The preliminary acute toxicity test showed that even at the highest dose of 360 mg/kg (iv), HGK-NSps had 100% of mice survival and all the mice were in a good state, suggesting a maximum tolerated dose more than 360 mg/kg. The effective antitumor effect and good tolerance showed HGK-NSps were likely to become a safe and effective antitumor drug for the treatment of breast cancer in the future.

## Introduction

Flavonoids come from a wide range of natural sources and have multiple biological activities, such as anti-inflammation, liver-protection, cardioprotection, neuroprotection, immunoregulation and so on (Perez-Vizcaino & Fraga, 2018), and thus consumed in large quantity worldwide as dietary supplements (Tungmunnithum et al., 2018). In addition, some flavonoids displayed mild inhibition against the growth of tumor cell lines.

**Figure 1.**
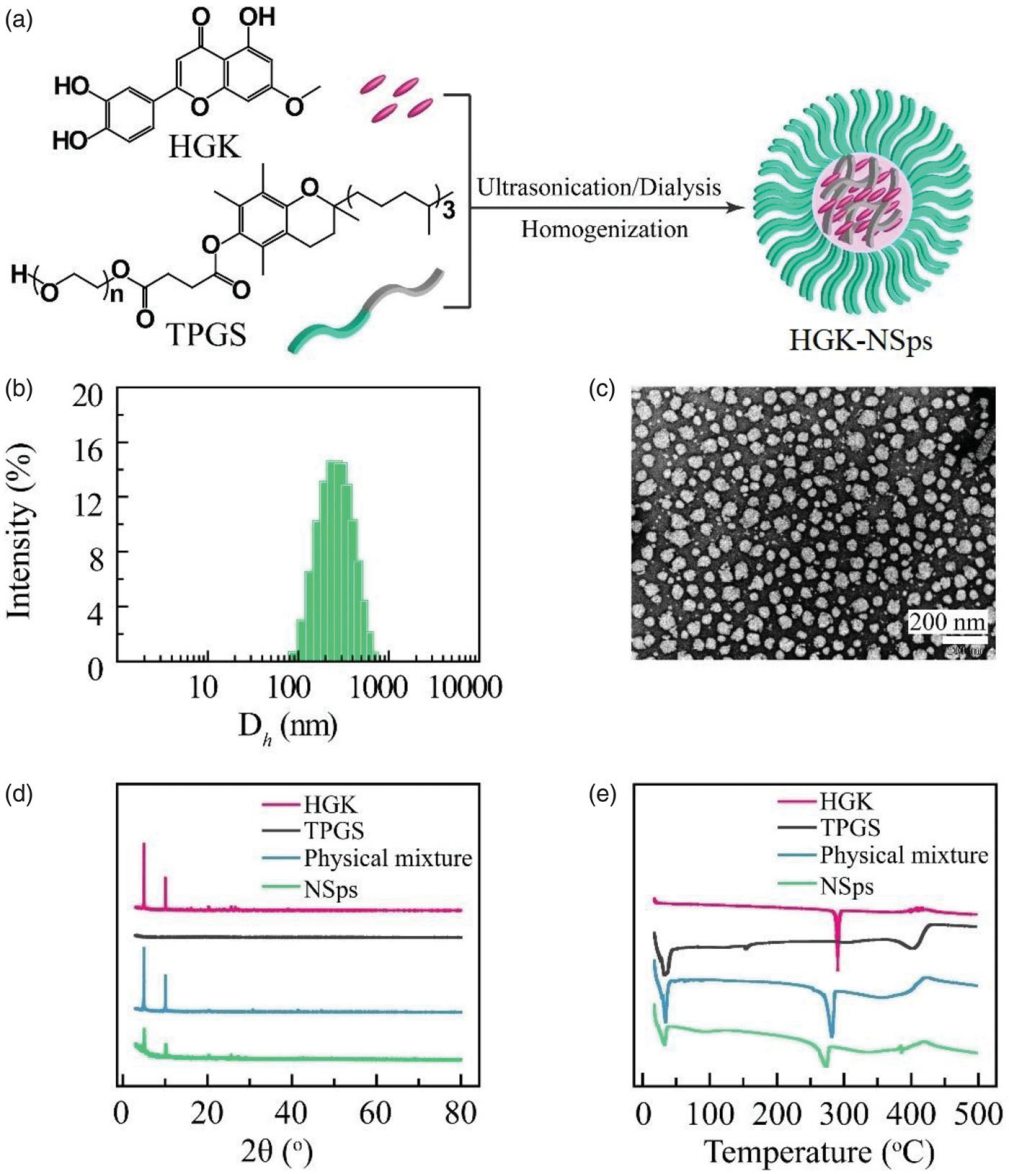
Chemical structure of HGK and characterization of HGK-NSps. (a) The chemical structure of HGK and TPGS, and the schematic illustration of HGK-NSps. (b) Particle size distribution of HGK-NSps (the feeding ratio of HGK/TPGS being 1:1). (c) TEM image of HGK-NSps. (d) XRD patterns. (e) Differential scanning calorimetry thermograms.

Hydroxy genkwanin (HGK, Figure 1(a)), with the molecular weight of 300.26 and molecular formula of C_16_H_12_O_6_, was firstly isolated from *Genkwa Flos* and then found in *Leonurus macranthus* and *Daphne tangutica Maxim* (Liu et al., 2009; Xie et al., 2011; Zheng H et al., 2017). It was reported that HGK showed antioxidant, immunomodulatory, antibacterial, and anti-inflammatory (Di Carlo et al., 1999; Wang et al., 2009; Xie et al., 2011; Yuan L et al., 2018). Due to its antioxidant activity, HGK can prevent thrombosis by inhibiting tissue factor (TF) (Jiang et al., 2009; Li N et al., 2008; Zhang et al., 2014). The only antitumor study was reported in 2013 by Wang et al. and in their work HGK inhibited the proliferation of glioma cells *in vitro* and showed synergistic inhibition with apigenin on C6 glioma cells (Wang et al., 2013). In our preliminary screening for new antitumor agents from natural resources, HGK was found to have very good antitumor activity against MCF-7 breast tumor and Hep3b liver tumor cells with IC_50_ value being approximately 4.9 µg/mL, while no cytotoxicity at all against normal HUVEC cells even at 20 µg/mL. But so far there has been no *in vivo* study performed to investigated the therapeutic efficacy of HGK on tumor-models. The partial reason behind may be that HGK is very insoluble in water (<1 μg/mL) and the pure compound of HGK is not easily available commercially in sufficient amount to support the *in vivo* study.

Nanotechnology and nanoscale drug delivery systems have been proved to be an effective way to solve the solubility problem and *in vivo* drug delivery of hydrophobic drugs. As one of the nanoscale drug delivery systems, nanosuspensions (Li et al., 2014) have gained extensive attention in the past decades due to their ability to load much more insoluble drugs than other nanoparticles and their suitability for various administration routes (Müller et al., 2001) and good biosafety (Wang et al., 2017). Nanosuspensions have the similar advantages characteristic of other drug-loaded nanoparticles, such as the oral availability enhancement, improvement of the *in vivo* pharmacokinetics and the significantly improved therapeutic efficacy for hydrophobic drugs. Several hydrophobic anticancer drugs have been prepared into nanosuspensions, including 10-hydroxycamptothecin (Pu et al., 2009; Zhao et al., 2010), honokiol (Han et al., 2014), annonaceous acetogenins (Hong et al., 2016), paclitaxel (Wang et al., 2019; Yin et al., 2016), with significantly improved the solubility, bioavailability, and the *in vivo* antitumor efficacy.

In this paper, HGK was successfully fabricated into nanosuspensions with good stability and significantly enhanced antitumor activity against 9 tumor cell lines. HGK-NSps displayed good tumor inhibition rate of 67.8% at 40 mg/kg and a maximum tolerated dose of more than 360 mg/kg, showing a good promise to be an effective but safe antitumor agent against breast cancer in the future clinic application.

## Materials and methods

### Materials

Hydroxy genkwanin (HGK) was supplied by Chengdu Herbpurify Co. Ltd (Chengdu, China). TPGS was provided by Xi’an Healthful Biotechnology Co. Ltd (Xi’an, China). mPEG_2k_-PCL_2k_ was purchased from Jinan Daigang Biomaterial Co. Ltd (Jinan, China). 3-(4,5-dimethylthiazol-2-yl)-2,5-diphenyltetrazolium bromide (MTT) and Pluronic F-127 (F-127) were obtained from Sigma Aldrich (St. Louis, MO, USA). Paclitaxel (PTX) injection was purchased from the Beijing union pharmaceutical factory (Beijing, China). All the other reagents were of analytical grade or higher. The water used in the experiments was deionized water.

### Cell lines and animals

The MCF-7 (breast carcinoma), BT474 (breast carcinoma), MDA-MB-231 (breast carcinoma), HepG2 (hepatic carcinoma), Hep3b (hepatic carcinoma), PLC/PRF/5 (hepatic carcinoma), SK-OV-3 (ovarian carcinoma), A549 (pulmonary carcinoma), HeLa (cervix carcinoma), and HUVEC (human umbilical vein endothelial cell) were supplied by China infrastructure of cell line resource. These cells were cultured with DMEM (MCF-7 cells, A549 cells, and HeLa cells), RPMI 1640 (BT474 cells), MEM (HepG2 cells, Hep3b cells, and PLC/PRF/5 cells), McCoy’s 5 A medium (SK-OV-3 cells), Leiboviz’s L15 medium (MDA-MB-231 cells), and Ham’s F12 medium (HUVEC cells), respectively, including 10% fetal calf serum (Thermo Fisher Scientific), streptomycin (100 U/mL), and penicillin (100 U/mL) with 5% CO_2_ at 37 °C (Sanyo, Osaka, Japan).

Female NU/NU nude mice (20 ± 2 g, 6-8 weeks old) and Kumingy mice (half male and half female, 20 ± 2 g, 6-8 weeks old) were obtained from Vital River Laboratory Animal Technology Co., Ltd. (Beijing, China). All animal experiments were conducted based on the Guidelines for Ethical and Regulatory for Animal Experiments as defined by the Institute of Medicinal Plant Development (IMPLAD), China. The ethics committee of IMPLAD granted ethical approval for this study and the approval number of the ethics committee was SLXD-20180514516.

### Preparation of HGK-Nanosuspensions

HGK nanosuspensions (HGK-NSps) were prepared by the combination of antisolvent precipitation and high-pressure homogenization. HGK powder and stabilizer (TPGS, F-127, mPEG_2k_-PCL_2k_, or Tween 80) were co-dissolved in N, N-dimethylformamide (DMF) as an organic phase (HGK concentration being 15 mg/mL). Subsequently, the mixed organic solution (0.2 mL) was dropped into 3 mL water at 25 ± 2 °C under 250 W ultrasonication. The mixture was then placed in a dialysis bag (MWCO: 8000–14000, Sigma, USA) and dialyzed under constant stirring (600 rpm) against 2 L of deionized water, which was renewed every half an hour for 4 hours to remove DMF. The obtained suspensions were homogenized under 1500 bar at 25 °C for 15 cycles to obtain HGK-NSps.

### Characterization of HGK-NSps

The mean particle size, polydispersity index (PDI), and zeta potential of HGK-NSps were detected by dynamic light scattering (DLS; Zetasizer Nano ZS, Malvern Instruments, UK) at 25 °C. The obtained HGK-NSps were diluted using deionized water to approximately 1 mg/mL HGK equivalent for use, and each measurement was performed in triplicate and with 12 runs. The morphology of HGK-NSps was investigated using a JEM-1400 transmission electron microscope (TEM; JEOL, Tokyo, Japan). A drop of HGK-NSps was dropped on the surface of the 300-mesh copper grid, air-dried, and dyed with 2% (w/v) uranyl acetate for observation under the electron microscope.

X-ray diffraction (XRD) measurements and differential scanning calorimetry (DSC) were applied to investigate whether the crystal structure of HGK changed during the preparation of nanosuspensions. An X-ray diffractometer (DX-2700, China) was applied to conduct X-ray powder diffraction with a generator set at 40 kV and 100 mA. Samples were scanned over an angular range of 3-80° of 2θ, with a step size of 0.02° and a count time of 3 s per step. Samples were rotated at 30 rpm during the analyses. DSC thermal profiles of the powder samples were tested through a differential scanning calorimeter (Q200, TA Instruments, New Castle, DE). Samples of approximately 10 mg were placed in standard aluminum pans, sealed with a lid and measured from 0 to 500 °C at a scanning rate of 10 °C/min under nitrogen atmosphere.

The HGK-NSps were lyophilized directly, the resultant dry powder was dissolved in methanol to disintegrate the HGK-NSps so as to completely release the drug into methanol solution, and then the HGK concentration in the methanol solution was measured by HPLC assay. The drug loading content (DLC) was calculated using the following formula:

(1) DLC (%) =V·C/W × 100%

where *V* is the volume of methanol, *C* is the concentration of HGK, and *W* is the weight of lyophilized powder of HGK-NSps.

### Chromatography analysis of HGK by HPLC

The HGK concentration in this study was determined by high-performance liquid chromatography (HPLC, DIONEX Ultimate 3000, USA) instrument. Chromatographic separation was conducted using a Symmetry C18 column (4.6 mm × 250 mm, 5 μm; Dr. Maisch GmbH, Germany) at 25 °C. The mobile phase consisted of water and methanol (15:85, v/v) and the flow rate was 1.0 mL/min. The detection wavelength of the UV detector was set at 337 nm. The standard curve equation was *Y* = 1.3679*X*-0.7747 (the linearity ranging from 0.1 μg/mL to 50 μg/mL, with *R*^2^ value of 0.9998).

### Stability of HGK-NSps in physiological media

HGK-NSps were, respectively, mixed with NaCl solution (1.8%), glucose solution (10%) and 2 × PBS (pH 7.4) (1:1, v/v), or mixed with plasma (1:4, v/v), followed by incubation at 37 °C. The particle size was measured at the set time. The above experiments were conducted in triplicates.

### Hemolysis test

Fresh rat blood was centrifuged at 3000 rpm for 5 min to obtain red blood cells (RBCs). RBCs were washed several times, diluted to 4% (v/v) with normal saline and then were mixed with normal saline (as negative control) and deionized water (as positive control) at the volume ratio of 1:1, respectively. HGK-NSps were mixed with 1.8% NaCl solution to obtain isotonic system and diluted to a series of concentration with normal saline, then mixed with prepared 4% RBCs suspensions (as tested groups) and deionized water (as blank control), respectively, at the volume ratio of 1:1. The mixtures were incubated at 37 °C for 4 h and then centrifuged at 3000 rpm for 5 min. The absorbance of the supernatant was detected at 540 nm. The hemolysis rate (*R_h_*) was calculated as follows:

(2) $$R_{h}\left(\%\right)\;=\;\left(A_{t}-A_{n}-A_{b}\right)/\left(A_{p}-A_{n}\right)\;\times \;100\%$$

where *A_t_, A_n_*, *A_p_* and *A_b_* are, respectively, the absorbance value of the test group, negative control, positive control, and blank control. Each sample was conducted in triplicates.

### *In vitro* drug release behavior

HGK-NSP (4 mL, 350 μg/mL) were placed in Float-A-Lyzer dialysis cassettes (MWCO: 8000–14000). Then, the dialysis cassettes were immersed into PBS (pH 7.4, 1 L) containing 5% (w/v) tween-80 and incubated at 37 °C with constant stirring (100 rpm). 50 μL internal dialysate was taken out from the dialysis cassettes at predetermined time, diluted by 10 times with methanol and centrifuged at 13000 rpm for 20 min, then the supernatant was injected for HPLC assay for HGK concentration. Each time when the internal dialysate was withdrawn, 50 μL fresh release medium was replenished. And the external release medium was replaced with a new one every 24 h. The above experiments were conducted in triplicates.

### *In vitro* cytotoxicity assay

The cytotoxicity of HGK-NSps was conducted against MCF-7, BT474, MDA-MB-231, HepG2, Hep3b, PLC/PRF/5, SK-OV-3, A549, HeLa, and HUVEC cells using the MTT assay. 150 μL cell suspensions at the logarithmic growth phase were seeded in 96-well plates (8000 cells/per) and incubated at 37 °C in 5% CO_2_ for 24 h. Then, HGK-NSps or HGK DMSO solution was diluted with culture medium and added into the wells, followed by incubation for 48 h and treatment with 20 μL of MTT solution (5 mg/mL in PBS) for 4 h. Subsequently, the medium was removed, 150 μL DMSO was added to each well and the absorbance was detected at 570 nm. The cell viability rate was calculated as follows:

(3) $$\text{Cell}\;\text{viability}\;\text{rate}\;\left(\%\right)\;={\;\text{OD}}_{e}/{\text{OD}}_{c}\times \;100\%$$

where OD_et_ and OD_c_ are, respectively, the mean optical density of the experimental group and control group.

### *In vivo* antitumor efficacy in MCF-7 tumor-bearing mice

0.2 mL MCF-7 cell suspensions (4.0 × 107 cells/mL) was subcutaneously injected into female nude mice at the right armpit. The mice were randomly divided into 5 groups (6 mice each) when the tumor volume reached 100 mm^3^, and injected via the lateral tail vein every two days with 0.2 mL normal saline (as negative control), 0.2 mL paclitaxel injection (8 mg/kg, as positive control), 0.2 mL HGK-NSps (10 mg/kg), 0.2 mL HGK-NSps (20 mg/kg), and 0.2 mL HGK-NSps (40 mg/kg), respectively. The dose of HGK-NSps was calculated according to the equivalent HGK. The mice weight and the volume of tumors were measured every 2 days for 14 days during the experiment period. All the mice were sacrificed by cervical vertebra dislocation and dissected 24 hours after the last dose to collect tumor tissues. The tumor inhibition rate (TIR) was calculated as follows:

(4) $$\text{TIR}\;\left(\%\right)\;=\;\left(1\;-{\;W}_{e}/W_{n}\right)\;\times \;100\%$$

where W_e_ and W_n_ are, respectively, the mean tumor weight of the experimental group and negative control group.

### The preliminary acute toxicity study of HGK-NSps using mice

The acute toxicity research was based on the Technical Guidelines for Acute Toxicity Experiment for Chemical Drugs, issued by the National Medical Product Administration of China (http://www.nmpa.gov.cn/gsz05106/11.pdf).

20 Kunming mice (half male and half female) were randomly divided into 2 groups and were intravenously injected with a single dose of normal saline (0.2 mL) and HGK-NSps (0.2 mL, 360 mg/kg) after an overnight fast with free drink. The toxic reaction and mortality were observed, and the symptom, occurring time, poisoning degree, death time of the toxic reaction were recorded as well as body weight and the general behavior for each mouse for 14 days.

### Statistical analysis

The statistical analysis among the different groups was conducted using IBM SPSS Statistics software, Version 19 (IBM Corporation, Armonk, NY, USA). A value of *p* < .05 was considered statistically significant.

## Results and discussions

### Preparation and characterizations of HGK-NSps

The stabilizer plays an important role in the successful preparation of nanosuspensions and could also affect the physiochemical property of drug-loaded nanosuspensions, including the drug-loading content, mean particle size, and storage stability. To select a suitable stabilizer for HGK-NSps, TPGS, Pluronic F-127, mPEG_2k_-PCL_2k_, and Tween 80 were tried as a stabilizer to prepare HGK-NSps under the same conditions via the anti-solvent nanoprecipitation method in this study. It turned out that Pluronic F-127 failed to be an effective stabilizer for HGK-NSps as obvious precipitation occurred once the organic phase was dropped into the aqueous phase. In cases of TPGS, mPEG_2k_-PCL_2k_ and Tween 80, homogenization proved to be an indispensable procedure to reduce the mean particle size of the obtained HGK-NSps below 300 nm (Table 1), among which, TPGS led to the smallest particle size (approximately 261.1 nm), narrow size distribution (PDI value of 0.12), and the highest zeta potential of –27.5 mV. So TPGS was selected as the stabilizer for the preparation of HGK-NSps in the subsequent study.

**Table 1.** Characterization and results of HGK-NSps (mean ± SD, *n* = 3).

Stabilizer	Drug/carrier ratio	DLS results			DLC (%)
		Size (nm)	PDI	Zeta (mV)	
Tween 80	1:1	295.1 ± 7.8	0.11 ± 0.04	–27.2 ± 1.1	n.d.
mPEG_2k_-PCL_2k_	1:1	274.1 ± 6.0	0.17 ± 0.03	–22.3 ± 1.9	n.d.
TPGs	1:1	261.1 ± 4.8	0.12 ± 0.01	–27.5 ± 1.5	39.9 ± 2.2
TPGS	1:2	256.0 ± 4.5	0.13 ± 0.01	–26.5 ± 0.8	24.3 ± 1.4
TPGS	1:3	259.1 ± 6.4	0.17 ± 0.03	–29.8 ± 0.5	16.9 ± 1.2

Note: n.d.: not determined.

To further reduce the particle size, HGK/TPGS weight ratio (1:2 and 1:3) was tried in addition to prepare HGK nanosuspensions, with the expectation that more stabilizer may lead to smaller particles. Table 1 shows that the feeding ratio of HGK and TPGS had no significant effect on the particle size of the resultant nanosuspensions. Since the highest drug loading content (39.9%) was obtained at the feeding ratio of 1:1, this ratio was selected for the preparation of HGK-NSps in the subsequent study.

Figure 1(b,c) shows the particle size distribution and the morphology of HGK-NSps. In Figure 1(b), HGK-NSps shows a mean hydrodynamic diameter of 261.1 ± 4.8 nm, while the TEM photo displayed a mean particle size less than 100 nm (Figure 1(c)). This is because DLS method measures a hydrodynamic diameter in a “wet” state where the PEG chain of HGK-NSps was hydrated and fully extended outward, thus showing a larger size than that in the “dry” and “shrinked” state as observed by TEM. The solubility of buck HGK powder in water was less than 1 μg/mL at 25 °C, however, the HGK concentration in HGK-NSps surpassed 2 mg/mL easily and the highest concentration reached 36 mg/mL in this study, which made it possible for HGK to be well delivered *in vivo* at a high dose.

As seen in Figure 1(d), the diffraction pattern of the lyophilized HGK-NSps showed diffraction peaks at the same diffraction angles as those of HGK bulk powders and the physical mixture, indicating that HGK maintained the same crystalline in the resultant nanosuspensions as in HGK bulk powder.

The similar result was observed in the DSC examination (Figure 1(e)), where both lyophilized HGK-NSps powder and the physical mixture exhibited a sharp endothermic peak at 282.1 °C, which was in agreement with the melting point and the endothermic peak of HGK bulk powder. This also proved the crystalline form of HGK was maintained during the preparation of HGK-NSps in this study.

### The stability of HGK-NSps in physiological media and hemolysis test

**Figure 2.**
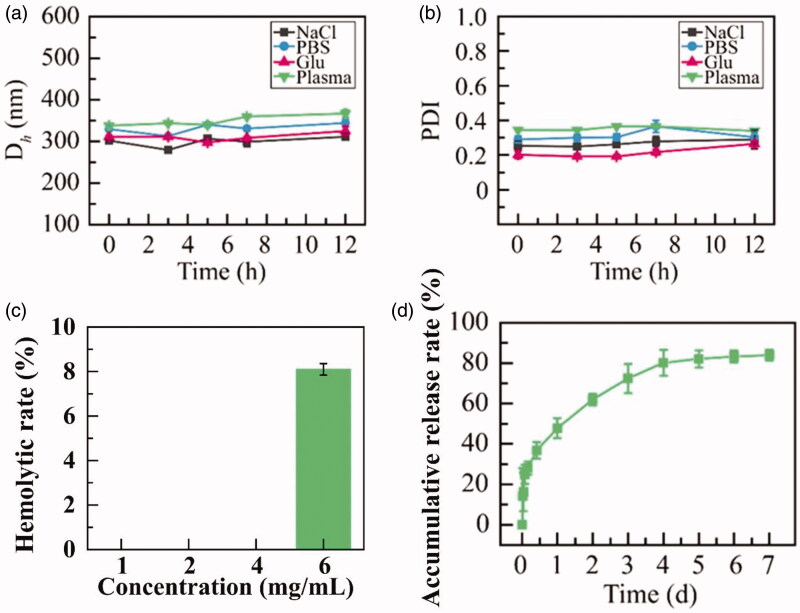
(a) Particle size change and (b) PDI value change of HGK-NSps during the incubation in normal saline, PBS, 5% glucose and plasma at 37 °C for 12 h. (c) The hemolytic rate and (d) *in vitro* drug release profiles of HGK-NSps (mean ± SD, *n* = 3).

Some nanosuspensions are stable in deionized water but unstable in PBS, normal saline or plasma with significant particle size augments or even aggregation and precipitation. The possible reasons for this phenomenon are in general ascribed to the effect of the ion strength, or the absorption of serum albumins on the surface of nanosuspensions. To make sure if similar phenomena occur to HGK-NSps, the stability of HGK-NSps in different physiological media, together with the hemolysis test, was studied. As seen in Figure 2(a,b), after incubation in normal saline, PBS, isotonic 5% glucose and plasma at 37 °C for 12 h, HGK-NSps maintained nearly the same particle size and PDI value in all the cases with no aggregation at all during the whole incubation process, suggesting the good stability of HGK-NSps in these physiological media and their good possibility for intravenous injection.

In the hemolysis assay, HGK-NSps were mixed with the same volume of 4% RBCs suspension and incubated at 37 °C for 4 h, and nearly no hemolysis was observed at the equivalent HGK concentration of 1, 2 and 4 mg/mL (Figure 2(c)), but there was less 8% of hemolysis for HGK-NSps at 6 mg/mL, showing HGK-NSps was safe for intravenous injection at low concentration.

### *In vitro* drug release behavior

As seen in Figure 2(d), approximately 28.1% of the encapsulated drug was released within the first 4 hours, which might be due to the relatively rapid release of a small amount of HGK encapsulated in the outer layer of HGK-NSps. Then there was a sustained drug release up to 80.1% till the 96th hour, followed by a very slow release up to 83.9% till the 168th hour.

Meantime, HGK suspension was prepared by dispersing HGK bulk powder into deionized water, and used as a control for the *in vitro* HGK release under the same conditions. However, no drug was detected during the whole period; this may be because that HGK was too hydrophobic to be released from the physical suspensions of HKG bulk powder under this condition. And this also showed that nanosuspension could be a good dosage form for HGK as nanosuspensions could well resolve its insolubility problem, significantly improve its *in vitro* release behavior of HGK, and expectantly realize the effective *in vivo* drug delivery.

### In vitro *cytotoxicity*

**Figure 3.**
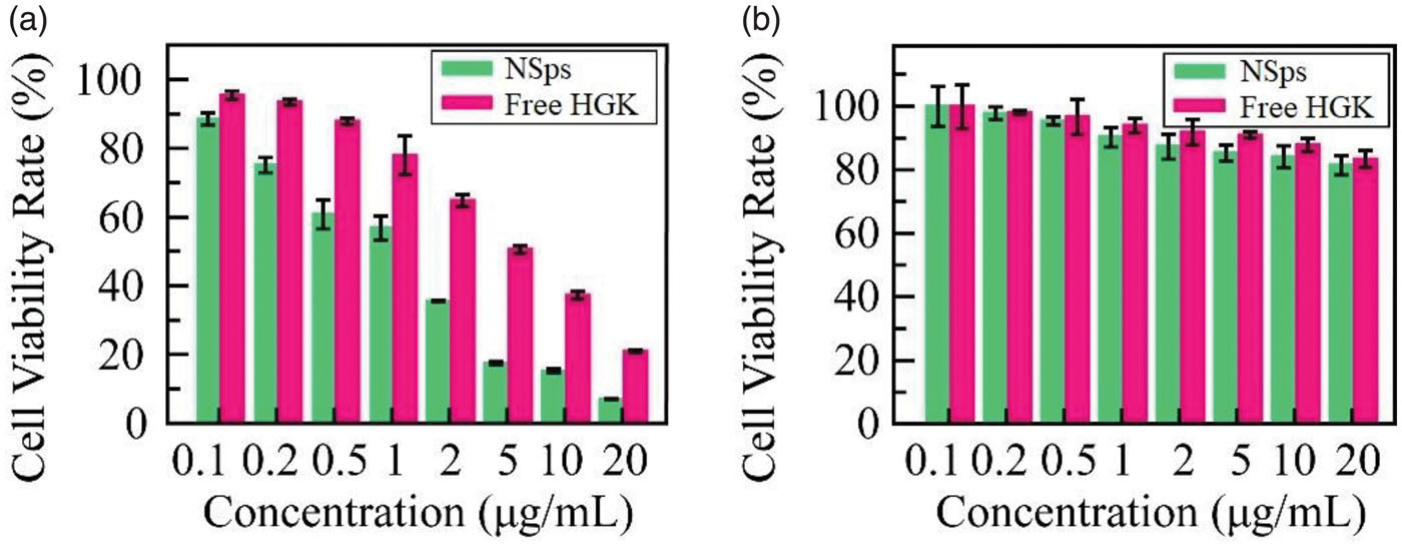
*In vitro* cytotoxicity of HGK-NSps and free HGK solution on MCF-7 cells (a) and HUVECs and (b) (mean ± SD).

The cytotoxicity of HGK-NSps against 10 cell lines, including MCF-7, BT474, MDA-MB-231, HepG2, Hep3b, PLC/PRF/5, SK-OV-3, A549, HeLa and HUVEC cells with free HGK (DMSO solution) as a control, was evaluated through MTT assay after 48 h incubation. Both HGK-NSps and free HGK suppressed proliferation in a dose-dependent pattern (MCF-7 as the example, Figure 3(a), see Figure S1 of supplementary materials for the data of other cell lines). But different cell lines showed different sensitivity to HGK and HGK-NSps. As listed in Table 2, both HGK-NSps and free HGK presented weak cytotoxicity against BT474 and A549 cells (the IC_50_ > 20 μg/mL), but strong cytotoxicity against the rest tumor cell lines, including MCF-7, Hep3b, HeLa, HepG2, PLC/PRF/5, SK-OV-3 and MDA-MB-231 with IC_50_ values being 1.0, 1.1, 2.1, 2.2, 3.3, 4.2, and 6.6 μg/mL, respectively. Besides, HGK-NSps showed significantly higher cytotoxicity than free HGK, IC_50_ values were reduced approximately 4.5-fold or more (4.9-fold for MCF-7, 4.5-fold for Hep3b, 6.3-fold for HeLa, 7.6-fold for HepG2, 15.1-fold for PLC/PRF/5, 5-fold for SK-OV-3, and 3-fold for MDA-MB-231, *p* < .01 in all cases). This may be because the encapsulation of HGK in nanosuspensions promoted HGK cellular uptake by these cell lines. It was reported that tumor cells can absorb nanoparticles nonspecifically and enhance their internalization through endocytosis and other mechanisms (Dong et al., 2016; Fernández-Urrusuno et al., 1996). In addition, HGK-NSps showed lower IC_50_ values against MCF-7 cell lines than other cell lines, which suggested that MCF-7 was most sensitive to HGK among these cell lines, thus MCF-7 bearing mice model was employed in the subsequent *in vivo* antitumor efficacy study.

**Table 2.** IC_50_ values of the HGK-NSps and free HGK solution against different cell lines after incubation for 48 h (mean ± SD).

Cell lines	HGK-NSps (μg/mL)	Free HGK (μg/mL)
MCF-7	1.0 ± 0.1**	4.9 ± 0.3
BT474	>20	>20
MDA-MB-231	6. 6 ± 1.0**	>20
HepG2	2.2 ± 0.4**	16.7 ± 3.0
Hep3b	1.1 ± 0.1**	4.9 ± 1.3
PLC/PRF/5	3.3 ± 0.7**	>20
SK-OV-3	4.2 ± 0.5**	>20
A549	>20	>20
HeLa	2.1 ± 0.4**	13.2 ± 2.4
HUVEC	>20	>20

**p < .01 vs. free HGK solution.

Besides these tumor cell lines, the cytotoxicity of HGK-NSps against normal endothelial cell lines HUVEC was also estimated (Figure 3(b)). Similar to free HGK, HGK-NSps showed no significant cytotoxicity to HUVEC, with the cell viability rate being above 80% at 20 μg/mL HGK equivalent. On the other side, the IC_50_ value of HGK-NSps against MCF-7 breast carcinoma cell lines was 20-fold lower than that of HUVEC cell line, normal cells in the body (1.0 vs. >20 μg/mL), which indicated that HGK-NSps may have certain selectivity and this may benefit the antitumor therapy.

### *In vivo* antitumor study

**Figure 4.**
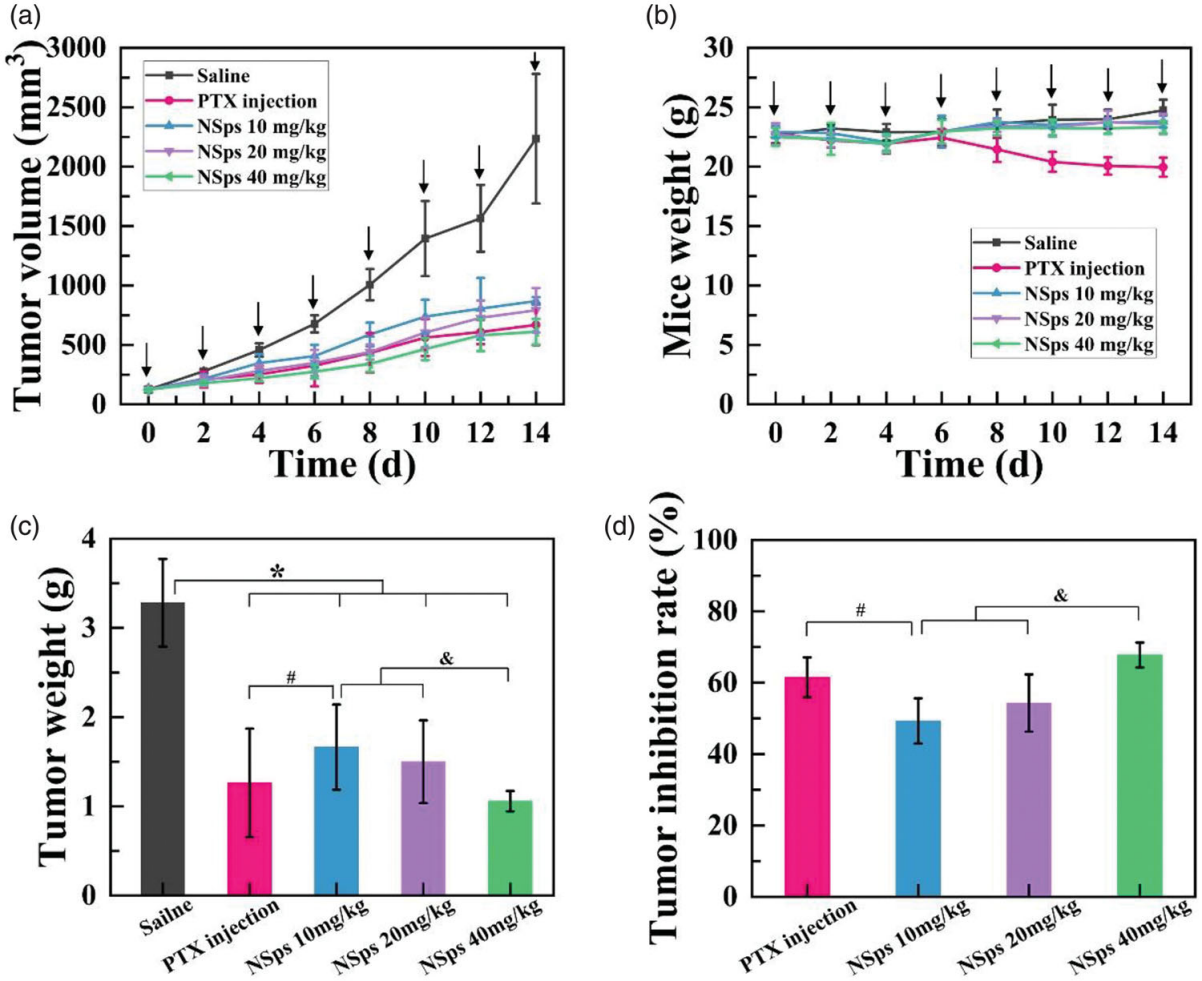
*In vivo* antitumor activity of HGK-NSps against MCF-7 tumor-bearing mice. (a) The tumor volume change curves. (b) Mice weight change. (c) Tumor weight *p < .05 vs. saline, #*p* < .05 vs. PTX injection, &*p* < .05 vs. NSps 40 mg/kg. (d) Tumor inhibition rate. For each animal, seven consecutive doses were given (marked by arrows). Data represent mean ± SD (*n* = 6).

**Figure 5.**
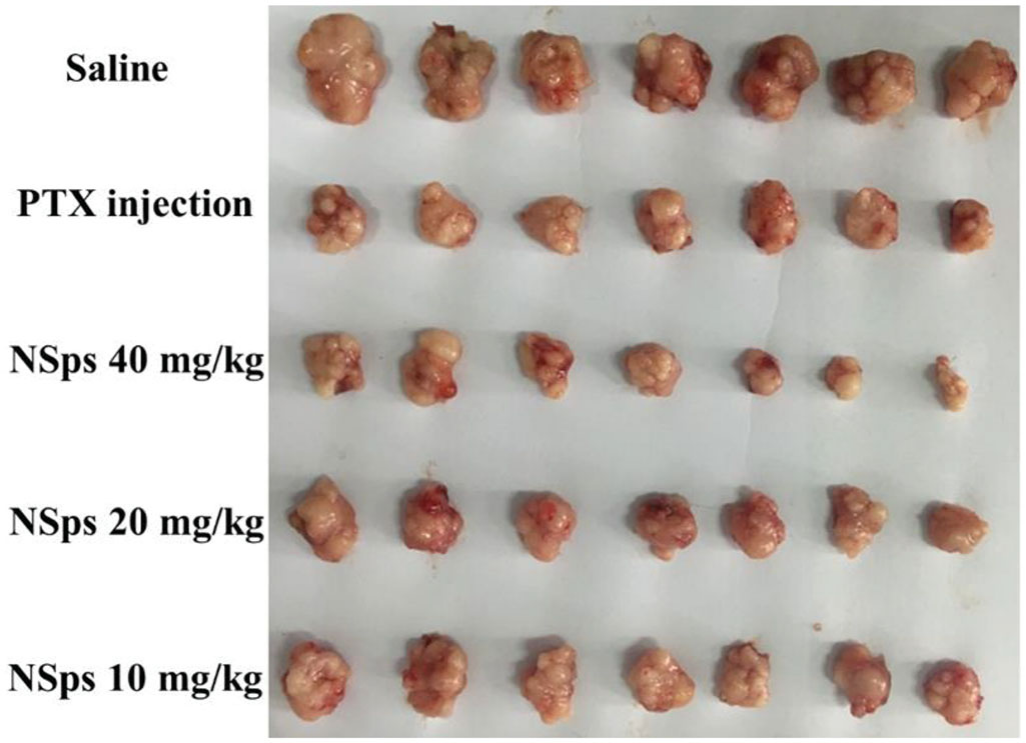
The actual photo of tumors for each group collected at the end of experiment.

The *in vivo* antitumor activity of HGK-NSps was investigated using MCF-7 tumor-bearing mice, and PTX injection was chosen as the positive control group due to its broad application in breast cancer (Demeckova et al., 2017; Eloy et al., 2017; Fujioka et al., 2017; Futamura et al., 2017; Zheng X et al., 2017). The tumor growth profiles and the bodyweight change were depicted in Figure 4(a,b), respectively. The average tumor weights and the tumor inhibition rates (TIR) were shown in Figure 4(c,d), respectively, with the actual tumor photograph seen in Figure 5.

During the whole process of the experiment, the mice in each group were all full of vigor with normal behavior. As shown in Figure 4(a), the tumor volume of the saline group rapidly increased to 2200 mm^3^ on the 14th day. On the contrary, limited tumor growth was displayed in both positive group (PTX injection) and tested groups (HGK-NSps), among which the high dose (40 mg/kg, iv) of HGK-NSps led to the least tumor volume growth, even less than that of PTX group (8 mg/kg), which was in good accordance with TIR calculated from the actual tumor weight (Figure 4(c)) measured at the end of the experiment. Both the tumor volume growth profiles (Figure 4(a)) and the TIR data (Figure 4(d)) displayed a clear dose-dependent antitumor efficacy for HGK-NSps, with TIR of 49.3 ± 6.3% for 10 mg/kg, 54.3 ± 8.0% for 20 mg/kg, and 67. 8 ± 3.5% for 40 mg/kg (*p* < .05, vs. 20 mg/kg or 10 mg/kg).

In addition, the *in vitro* biodistribution of HGK-NSps in MCF-7 tumor-bearing mice was also examined. As seen in Figure S2 in the Supplementary material, HGK-NSps mainly distributed in the liver and spleen after intravenous injection and barely benefited from enhanced permeability and retention (EPR) effect that may lead to more drug accumulation in tumor (Li M et al., 2020) (Hong et al., 2016; Jain, 2005; Yuan Z et al., 2015). The reasons behind were probably related to the large particle size or the specific surface property or the interior structural property of HGK-NSps.

It was verified that high dose of HGK-NSps had similar, if not better, antitumor efficacy in comparison with PTX injections (67.8% vs. 61.5%, *p* > .05, TIR), but there was very significant different in their body weight change (Figure 4(b)). For an antitumor agent, systemic toxicity is another important point to evaluate during tumor treatment. Bodyweight and median lethal dose (LD50) are common indicators for assessing systemic toxicity. The body weight growth curves of the three HGK-NSps groups were close to that of normal saline group while the PTX group showed significant bodyweight reduction after the fourth dose (Figure 4(b)). This indicated HGK-NSps may have better safety than PTX when used in clinic.

### The preliminary acute toxicity

In the preliminary acute toxicity trial, 10 Kunming mice were intravenously injected with HGK-NSps at 360 mg/kg (single dose), the highest HGK equivalent can be achieved for HGK-NSps, with another 10 Kunming mice intravenously injected with 0.2 mL normal saline as a control. Only three male mice experienced a short time of curling up, turning over or hypokinesia immediately after dose and then recovered quickly to the normal state. The rest mice were full of vigor with normal saline, as did in normal saline group. During the 14 days of observation, there was no death, no abnormal behavior for all the mice, and the HGK-NSps group showed nearly the same bodyweight growth profile as that of normal saline group. This meant the maximum tolerated dose for HGK-NSps (iv) was more than 360 mg/kg and the LD50 was much more than 360 mg/kg, at least 9 times of the therapeutic dose, showing very good safety and prospect of HGK-NSps in clinic use. The main reason behind may be related to the fact that HGK was not a wide-spectrum antitumor agent, but selectively suppress MCF-7 breast tumor and Hep3b liver tumor. Since the survival rate was 100% at the highest dose achieved, further acute toxicity trial failed to be performed.

## Conclusions

In the long run, it is still an important task for us to find a new but effective and safe antitumor drug due to the serious side effect and the recurrent multi-drug resistance of the current chemotherapeutics used in the clinic. In our screening for new antitumor agents from natural resources, HGK was found to have good antitumor activity against MCF-7 breast tumor and Hep3b liver tumor. In this paper, we successfully improved the solubility of HGK through nanosuspensions and carried out the antitumor research. Using amphiphilic TPGS as a stabilizer, HGK-NSps were prepared with high drug loading content, good stability in various physiological media, and sustained *in vitro* drug release. HGK-NSps exhibited a more effective proliferation inhibitory effect than free HGK against a series of tumor cell lines, among which Hep3b, MCF-7 and Hep3b were most sensitive. *In vivo* studies using MCF-7 tumor-bearing mice suggested that HGK-NSps (40 mg/kg) could achieve similar therapeutic efficacy in contrast with PTX injection (8 mg/kg), but better safety on basis of body weight change. Even at the highest dose achieved (360 mg/kg), mice showed good tolerance to HGK-NSps with 100% of the survival rate in the acute toxicity trial, probably due to the selective antitumor effect of HGK. The effective antitumor action and good tolerance showed HGK-NSps were likely to become a safe and effective antitumor drug for the treatment of some breast and liver tumor in the future. To our knowledge, this is the first report the first time that HGK was prepared into nanoparticles and showing antitumor activity of HGK *in vivo*.

## Supplementary Material

Supplemental Material
