# Supplementary material for: Preparation of hydroxy genkwanin nanosuspensions and their enhanced antitumor efficacy against breast cancer
Source: Drug Deliv. 2020 Jun 3;27(1):816–24. doi: 10.1080/10717544.2020.1770372 (PMC8216440; doi:10.1080/10717544.2020.1770372)
Supplement: Supplemental Material [file IDRD_A_1770372_SM0992.docx]

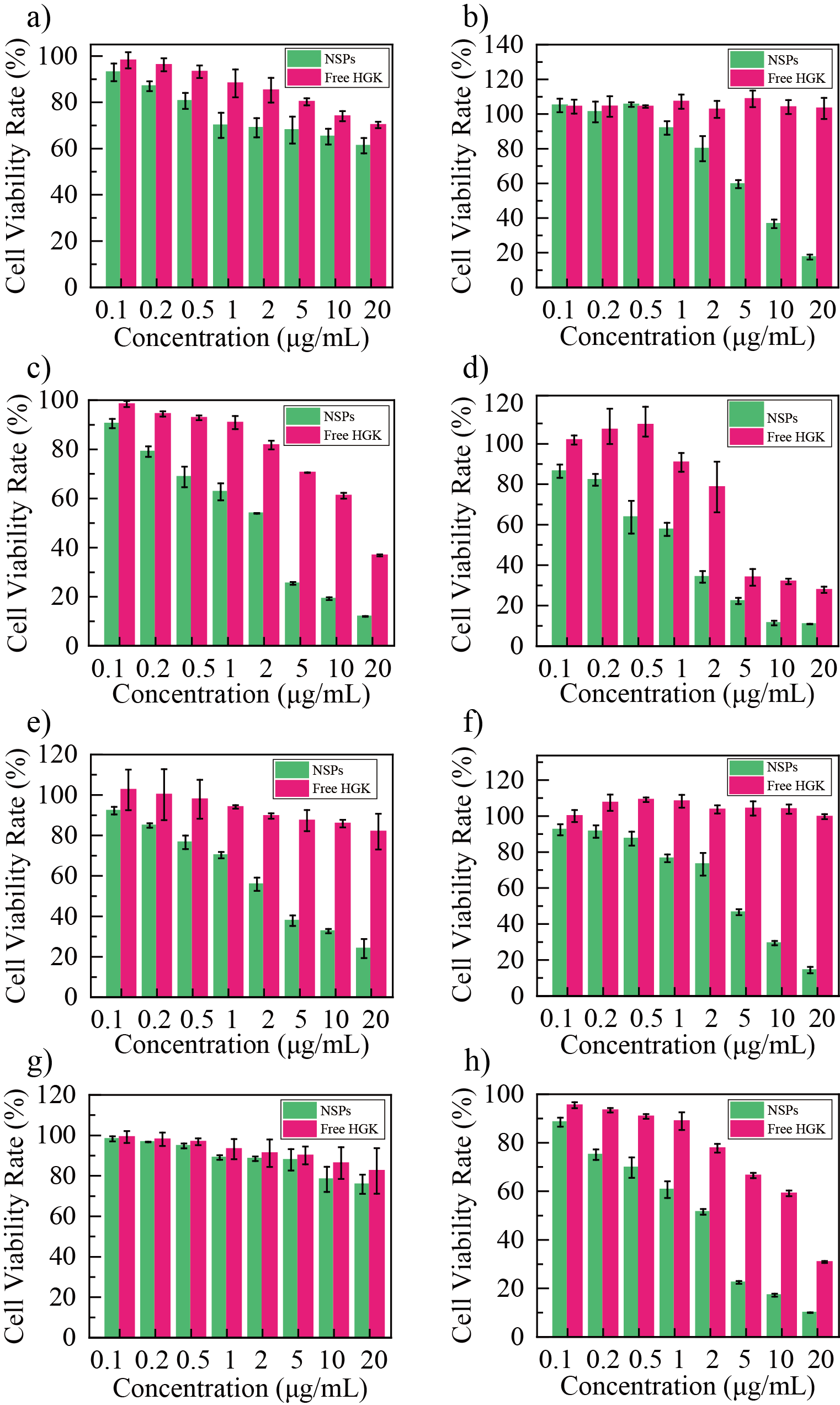


**Fig. S1** In vitro cytotoxicity of HGK-NSps and free HGK solution on (a) BT474 cells, (b) MDA-MB-231 cells, (c) Hep G2 cells, (d) Hep3b cells, (e) PLC/PRF/5 cells, (f) SK-OV-3 cells, (g) A549 cells and (h) HeLa cells (mean ± SD).


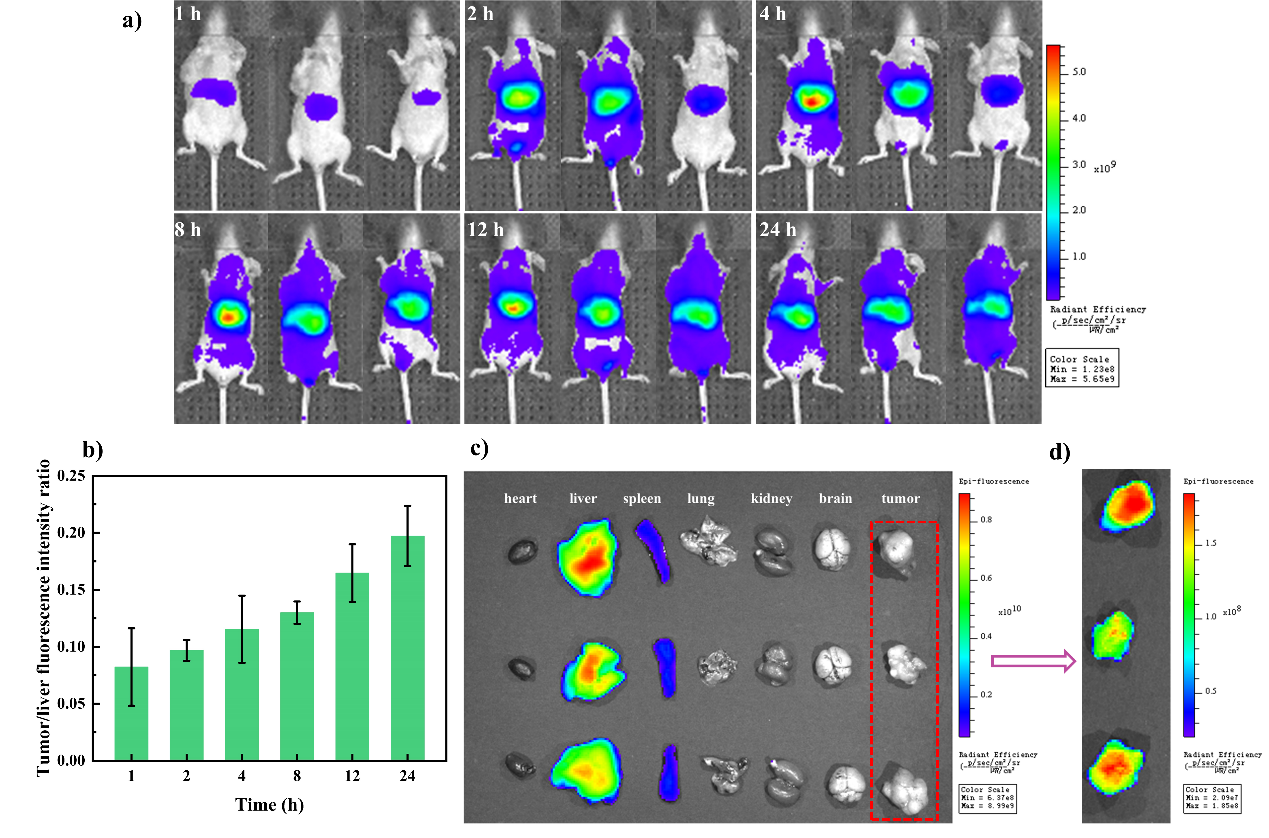


**Fig.S2** In vivo biodistribution of HGK-NSps in MCF-7 tumor-bearing mice: (a) dynamic biodistribution of HGK-NSps at different time. (b) tumor / liver fluorescence intensity ratios at different time. (c) biodistribution of HGK-NSps in isolated organs 24 h after administration. (d) biodistribution of HGK-NSps in tumor 24 h after administration.

Method

In vivo biodistribution in MCF-7 tumor-bearing mice

DiR, a lipophilic fluorescent dye, can exhibit a specific color at a specific wavelength. In order to visually observe the distribution of HGK in MCF-7 tumor-bearing mice, HGK-NSps loaded DiR was prepared as the method “Preparation of HGK-NSps”, except that HGK was replaced by a mixture of HGK and DiR (HGK : DiR = 40: 1, w / w). 0.2 mL MCF-7 cells suspensions (4.0 ×10^7^ cells/mL) was subcutaneously injected into female nude mice at the right armpit. When tumors size reached about 500 mm^3^, tumor-bearing mice are injected intravenously with HGK/DiR-NSps (40 mg/kg) and were imaged using IVIS Living Image^@^ 4.4 (Caliper Life Sciences, Hopkinton, MA) at a set time after administration. After 24 h, the mice were sacrificed, and the tumors and major organs were excised, then were imaged as described. Living Image software (version 4.2) was used for quantitative analysis.
